# Disrupted Sleep at Night Is Associated with a Higher Level of Agitation Throughout the Week in People Living with Cognitive Decline

**DOI:** 10.1002/alz.090591

**Published:** 2025-01-03

**Authors:** Wan‐Tai M. Au‐Yeung, Lyndsey M. Miller, Allison Lindauer, Zachary T Beattie, Nora Mattek, Joel S. Steele, Jeffrey A Kaye

**Affiliations:** ^1^ Oregon Center for Aging & Technology (ORCATECH), Portland, OR USA; ^2^ Oregon Health & Science University, Portland, OR USA; ^3^ NIA‐Layton Aging & Alzheimer’s Disease Research Center, Portland, OR USA; ^4^ University of North Dakota, Grand Forks, ND USA; ^5^ Oregon Health & Science University, Oregon Center for Aging & Technology (ORCATECH), Portland, OR USA

## Abstract

**Background:**

Agitation is one of the most challenging behaviors exhibited by people with cognitive decline, causing distress in caregivers, earlier placement into long‐term care and faster disease progression. In order to better manage agitated behaviors in people with cognitive decline, it is important to identify associated factors. The MODERATE (Monitoring Dementia‐Related Agitation Using Technology Evaluation) study aims to characterize agitation using technology and identify precipitants (behavioral or environmental) of agitation.

**Method:**

People living in the greater Portland, Oregon, USA area were recruited into the study. The inclusion criteria were a diagnosis of mild cognitive impairment or dementia, currently residing with a family caregiver within their own home, and caregiver endorsement of symptom(s) of agitation, irritability/lability, motor disturbance, nighttime behaviors and/or disinhibition in the Neuropsychiatric Inventory‐Questionnaire (NPI‐Q). Bed pressure mats (Emfit) were deployed as part of the ORCATECH platform in their homes, which measure participants’ sleep behaviors and their physiological signals through ballistocardiography. The weekly agitation levels of the participants were reported by their family caregivers through modified Cohen‐Mansfield Agitation Inventory‐Short Form questionnaires as part of weekly online surveys.

**Result:**

Data from the first 8 participants (mean age(SD) = 70 (10)) with bed pressure mats were analyzed. A subset of bed pressure mat outputs was selected to provide a holistic picture of participants’ sleep behaviors and their physiology while avoiding multicollinearity. Monitoring period was for a mean (SD) of 315 (190) nights. An individual mixed linear model was built with each included bed pressure mat output and week as the predictors and total agitation level as the model output. After false discovery rate correction, higher duration of wake after sleep onset was a significant predictor of their weekly total level of agitation. The caveats of these results include small sample and varied follow‐up time ranging from 4‐85 weeks of observations per participant.

**Conclusion:**

Disrupted sleep (measured objectively by bed pressure mat) are positively associated with participants’ weekly level of agitation. Future work with larger sample sizes is needed to confirm findings. Unobtrusive identification of behavioral factors associated with agitation can potentially help manage this challenging behavioral symptom.

